# The Hospital. Nursing Section

**Published:** 1904-12-03

**Authors:** 


### The Hospital.

### Nursing Section.

Contributions for this Section of "The Hospital" should be addressed to the Editor, "The Hospital," Nursing Section, 28 & 29 Southampton Street, Strand, London, W.C.

No. 949.—Vol. XXXVII. *SATURDAY, DECEMBER 3, 1904.*

### Notes on News from the Nursing World.

### HONOURS FOR A SCOTTISH NURSE.

The King has presented the Victorian Medal to Mrs. Bell Edgar in recognition of her services as nurse to the Duke of Connaught since his motor accident. She has also received from the Duke and Duchess of Connaught a pearl brooch in acknowledgment of her care and attention to the Duke as well as a testimonial of an appreciative character. Mrs. Edgar, who is the widow of Mr. Bell Edgar, formerly Burgh Prosecutor for Moffatt, was trained at the Royal Infirmary, Glasgow, and is a member of the Scottish Association of Trained Nurses.

### READ THE LABEL.

The vital importance of a nurse always and under all circumstances carefully reading the label of any bottle out of which she administers a dose to a patient, has again been illustrated in a most painful manner. In the course of an inquiry at the Liverpool Police-court last Saturday, concerning the death of a marine fireman who died on the previous Thursday at the Stanley Hospital, it transpired that one of the nurses—not a raw probationer, but a nurse who has been in the hospital for upwards of three years—gave the deceased a dose of caustic soda instead of a dose of magnesia, and although the medical evidence was to the effect that the deceased would probably not have lived even if he had not swallowed the caustic soda, the jury returned a verdict that death was due to misadventure on the part of the nurse. They added a recommendation that all poisons should be labelled and kept in a separate place. The recommendation is more than justified by the facts disclosed, which showed conclusively that poisons and aperients were kept on the same shelf in similar bottles without even the red poison label to distinguish them. For this discreditable state of things the hospital authorities are to blame. But the nurse who took a bottle off the shelf which she "thought was the aperient" had no business to think. Her business was to read the label, whether its colour was red, or white, or black; and if she had done so she would have discovered that it was caustic soda at the time, instead of finding her error out after the mischief was perpetrated.

### MIDWIVES AND MORAL CHARACTER.

At the seventh annual meeting of the Sussex County Nursing Association, which is affiliated to Queen Victoria's Jubilee Institute, and has just federated with the Royal National Pension Fund, Miss Amy Hughes, referring to the statement of an eminent medical authority that the question of moral character did not enter into a midwife's work, said she thought "that it was most necessary that midwives should be women whose character and influence made for good." Miss Hughes is perfectly right. The Midwives Act will, we hope, tend to ensure not only the employment of properly-trained women, but also of persons of high moral character. Of course, registration merely guarantees that they are technically qualified for their work, but those whose character is known to be above suspicion will be more likely to secure the confidence of the public.

### SOUTHWARK GUARDIANS AND RELIGIOUS TESTS.

The choice of a superintendent nurse by the Southwark Guardians was made the occasion of a discussion on the subject of religious tests. Some of the guardians insisted that the most suitable candidate had not been recommended by the Newington Visiting Committee because she is a Roman Catholic. If that be so, an injustice has been done. But the guardians who voted for Miss Florence Collins declare that she was chosen because of her professional qualifications, and not because she is a Protestant. The dissentients being in a minority, notice has been given of a proposal "that in future appointments for any post under this board no question respecting religion be put." We think that so long as the guardians are permitted to put questions at all, it is quite natural that they should ask one respecting religion. Moreover, if they were hindered from asking it point blank, they would probably be able to obtain the information in another way. Our objection is not to the question, but to any attempt to exclude from any position in nursing under the Poor Law any person merely on account of her religion.

### THE NURSE-STEWARDESS.

The question of employing trained nurses at sea, which is an old one so far as this paper is concerned, is being discussed in the leading Indian journal. It appears to be thought a matter for great congratulation that the Peninsula and Oriental Steamship Company give a preference in the selection of their stewardess to persons who have had hospital training. In our opinion this is no solution of the question, and it has been shown by correspondents writing to us in the past that the attempt to combine the duties of stewardess and nurse inevitably ends in failure. In the first place a stewardess is not treated by the passengers in the manner in which a trained nurse expects to be treated, and in the second, if a nurse is to be of any real value on board a liner, she must be able to give her undivided attention to her patients. It is the general experience that the stewardess is fully occupied with the ordinary work of her department, and we do not see how it is possible for her to try to act as a nurse without neglecting it. The idea of the nurse-stewardess may afford gratification to managers anxious to obtain credit for studying the interests of the public without expense to the pockets of the companies they represent; but in practice the arrangement is a miserable makeshift for the proper and necessary course of employing a trained nurse under precisely the same conditions as she is usually employed.

### THE PHYSICALLY UNFIT NURSE.

The sad suicide the other day of a young woman who desired to be a nurse, and on her examination at a London fever hospital was found to be physically unfit for the work, must be regarded as at once a serious mistake on the part of the provincial doctor who had given her a certificate of fitness, and a complete vindication of the decision of the medical authority at Stockwell. A doctor never refuses to pass a candidate for the post of probationer if he can conscientiously say that she is physically able to undertake the work. But we are afraid that in some cases, either in consequence of excessive optimism, or of want of proper attention, unsuitable persons are not rejected as they should be. We should hear less about delicate and tired nurses, less, too, about indifferent nurses, if all medical officers declined to pass any one not possessing an absolutely sound constitution.

### INCREASE OF PAUPER ATTENDANTS IN THE NORTH.

In his nursing return for the counties of Lancaster, Westmorland, and part of Cumberland, Mr. Jenner-Fust reports that there has been an increase of 41 in the number of nurses employed, the number of patients per nurse being 11·28 on January 1st, 1904, as against 12·66, 12·73, and 12·74 on January 1st, 1903, 1902, and 1901 respectively. During the nine years since the return was first issued the number of patients per nurse has diminished from 17·48 to 12·28; and whereas nine years since the return was first issued the number of night nurses employed was only one to 81 patients, it is now one to 48. This, we agree with Mr. Jenner-Fust, is a great advance, but still further improvement is very much required. It is an unsatisfactory feature that the number of pauper attendants in the district shows a slight increase; nothing but a steady decrease, until at length pauper attendants are utterly swept away, can be satisfactory.

### THE ENGLISH NURSE ABROAD.

In our issue last week there was a letter from "An English Nursing Sister," who laid down a high standard for private nurses working abroad. This standard, unfortunately, is often not reached, and the result is that in some foreign cities English nurses do not inspire confidence. In Rome, we are informed, many of them have inspired feelings of a very different character. Their rivalries, jealousies, and inability or indisposition to work in harmony are said to have been a matter of common remark. We greatly regret to hear this. The existence of an institution which is not managed satisfactorily is not a reason why individual nurses should neglect to observe the first traditions of their vocation.

### A RETROGRADE PROPOSAL AT NORTHALLERTON.

Because there are at present only six patients in the Northallerton Workhouse Infirmary, some of the guardians are urging that the services of a nurse should be entirely dispensed with. The proposal was made at the last meeting in spite of an entry in the medical relief book certifying that two inmates were unfit to be left at night without a night attendant. In any event, in cases where the matron herself has not been trained, the appointment of a trained nurse is essential, and an attempt to do without one would call for the immediate intervention of the Local Government Board.

### STRANGE ABSENCE OF DISCIPLINE AT AN IRISH INFIRMARY.

The two nurses at Thurles Workhouse Infirmary who were reprimanded by the Guardians, as stated in our issue last week, have since sent letters denying some of the charges made against them, and asking for the rescission of the order. Their letters have been marked by the Guardians as "read," but the latter have declined to reopen the question. The nurses could have made their statements at the time they were desired to appear before the Guardians, and, as they refused to take advantage of the opportunity, we do not think that in all the circumstances they have any cause for complaint.

### QUEEN ALEXANDRA'S MILITARY NURSING SERVICE.

The following staff nurses belonging to Queen Alexandra's Imperial Military Nursing Service have been appointed sisters:—Miss M. L. Harris, Miss A. M. Harrison, Miss J. E. Mackay, Miss E. S. Mason, and Miss M. Walker. The appointments of Miss L. M. Moir and Miss M. E. Richardson as staff nurses have also been confirmed.

### A NEW DEPARTURE AT THE NORTH DEVON INFIRMARY.

The House Committee of the North Devon Infirmary have decided to start a private nursing staff in order to supply the public with the services of trained nurses. One nurse has already been engaged. The experiment appears to be much approved in Barnstaple, and the new departure should prove useful to the neighbourhood. The terms on which nurses will be supplied are: medical and surgical cases, £1 10s. per week or part of a week; mental, nervous, and infectious cases, £2 2s.; monthly cases, from £6 6s. per month. For shorter periods than a week, or for attendance at operations, the charge is 10s. 6d. per day. If the nurse is required to sleep in the infirmary, instead of the patient's house, 10s. a week extra is charged; and the limit of attendance for the same nurse upon a case is eight weeks, unless by special permission of the committee.

### "SICK-ROOM HELPS" SOCIETY.

A drawing-room meeting on behalf of the "Sick-Room Helps" Society, whose objects are maternity nursing and the care of the home among the Jewish poor in the East End of London, will take place on Monday at 25 Park Lane, Lady Sassoon in the chair. We observe, from the tenth annual report of the Society, that the total income was £1,842 10s. 7d., and the total expenditure £1,830 9s 5d. Included in the latter sum is £155 4s. 6d. which represents the investment of capital, but as the subscriptions and donations were under £700, a considerable increase on this amount is needed to supplement adequately the contributions from the poor mothers. The staff now consists of five qualified nurses and 36 helps. The report includes a passage to the effect that a great increase in the number of Jewish district nurses is urgently required.

### QUADRILLES AND DISTRICT NURSING.

The Bridport Nursing Society appears to have been saved during the past year from financial disaster by the help of a quadrille party. The subscription list for the 12 months showed a falling off, and the church and chapel collections were smaller than they were in the previous period. But as the Mayor said in his speech at the annual meeting, the Committee of the Private Quadrille Party Ball came to the rescue with most generous assistance and their donation of £11 9s. 6d. made all the difference to the Association. The Mayor expressed a hope that the Committee would be able to hand over something more in the future, while the rector of Bridport said that he thought all the places of worship in the town might endeavour to arrange collections for the fund. Even so, however, it is important that the list of regular subscribers should be augmented, and that the managers of the Association should be in a position to reckon upon this source of income for the payment of ordinary expenditure.

### GUARDIANS AND QUEEN'S NURSES.

The Local Government Board have been asked to sanction a subscription by the Ecclesall Guardians of £200 to the Sheffield Branch of the Queen's Jubilee Institute for a further year, and an additional £50 to cover the expenses in connection with providing all the medical necessaries for the poor within the union. The request is based on the ground that out of the 686 patients attended by the Queen's Nurses during the first year of the existence of the Association 94 were resident poor in the union and in receipt of out-door relief. The guardians are well represented on each Committee of the Association, and the relations between the two bodies appear to be all that could be desired.

### RETROGRESSION AT BRIDLINGTON.

There ought to be no difficulty in such a prosperous watering place as Bridlington in obtaining adequate funds for the support of two nurses. According to the fifth annual report of the Bridlington and District Nursing Association, however, the balance in hand at the end of the financial year has been reduced by half, and this, too, in spite of the fact that the patients themselves contributed £4 8s. more than in the previous 12 months. The practical appreciation of the services of the nurses thus shown by the patients should act as an incentive to the generosity of the residents. The record of the year's work is 3,260 visits paid and 206 patients nursed. The association seems to be admirably managed, but if the committee want to keep a balance in hand, they will evidently have to urge increased energy upon their collectors.

### SOCIAL FUNCTION AT PORTSMOUTH INFIRMARY.

A very pleasant evening was spent on Saturday last by the nursing and medical staff of the Parish of Portsmouth Infirmary on the occasion of the first nurses trained in midwifery at this school gaining the certificate of the London Obstetrical Society. The large recreation-room was elaborately decorated with gold and white, yellow and white chrysanthemums in abundance forming the floral decorations. Between the dances solos and duets were rendered by some of the nurses, the medical superintendent, and his assistant. The latter were greatly helped in their efforts to make the evening enjoyable by the matron, who, although she has only been at Portsmouth a short time, has endeared herself to the staff by her kindness and tact.

### A NURSING ASSOCIATION FOR BLACKPOOL.

It has been decided to form a Nursing Association at Blackpool. The meeting convened for the purpose entered with spirit into the proposal, and it was decided that the town should be divided into districts to be canvassed for subscriptions. A fair sum of money was promised on the spot, and officials have since been appointed. More than a dozen ladies have taken charge of districts.

### OUR CHRISTMAS DISTRIBUTION.

As the date of our Christmas distribution of clothing approaches the anxiety to participate in it becomes more keen. For instance, the matron of a well-known hospital in a very poor locality writes to us:—"Last year you were kind enough to send to this hospital a beautiful gift of clothing as a Christmas gift. The garments were so needed and I was so grateful that I venture this year to ask if I may look for a similar present. We are situated in a very poor district, and I find it very hard work to get a few presents ready for our patients at Christmas time." The severity of the weather is augmenting the distress usual at this period of the year, and there is no doubt that the needs of the sick poor in hospitals for warm clothing will be greater than they were last year. We have received contributions from Miss Orme, The Lodge, Appleby Castle, Westmorland, and Miss Henslow, Carrington Close, Weymouth. All parcels should be addressed to the Editor, 28 & 29 Southampton Street, Strand, London, W.C., with "Clothing Distribution" written on the outside.

### SHORT ITEMS.

The Scientific Press announces that they will shortly publish an Elementary Treatise on the Light Treatment for Nurses, by Dr. James H. Sequeira, physician in charge of the Skin department of the London Hospital.—The handsome sum of £123 was realised by a sale of work in York on behalf of the District Nursing Fund extending over two days.—It has been decided to engage a trained nurse for the district of St. Blazey and St. Mary's, Par, Cornwall, and an association has been formed with a representative committee. The salary of the nurse has been fixed at £60 per annum.—A movement has been set on foot to secure a Queen's Nurse for the villages of Mangotsfield and Pucklechurch, near Bristol, and the necessary committee has been elected.

### The Nursing Outlook.

"From magnanimity, all fear above;
From nobler recompense, above applause,
Which owes to man's short outlook all its charm."

### WOMEN AS HOSPITAL MANAGERS?

The suitability of women for seats on hospital committees has periodically formed the subject of debate for years. Under the presidency of the Dowager Countess of Shrewsbury the question has once more been revived and a conference held. Miss Georgiana Hill, who opened the discussion, declared that at present women are either excluded, or only admitted to a certain extent on the management of hospitals of inferior standing. Women have nothing whatever to do with the management of great hospitals like Guy's and the London, and there are 50 other hospitals in London which do not possess any ladies' committee. Such is Miss Hill's statement. She expressed much indignation at this state of affairs, and Lady Shrewsbury's conference advocated that women should refrain from supporting hospitals until they secured control in their management.

We gather in the first instance from the line taken by Miss Hill and her supporters that they are not familiar with the inner working of a great hospital. They do not seem to possess sufficient practical knowledge to enable them to differentiate between one hospital and another, or to understand that whereas the presence of women may be very helpful in certain cases, it may be undesirable and even objectionable in others. At a great hospital, especially one which is connected with a medical school, the whole of the work has to be systematised, and its volume is often so great that everything connected with the establishment should be managed with the precision of a business, or efficiency must suffer. As a fact, in all the large hospitals, women do exercise a very active and controlling influence on the management, because their representatives are ladies of experience, knowledge, and training, who devote the best years of their lives to the work. Of course the women who work in the great hospitals are paid for their services. This fact does not in any way diminish their value, but, on the contrary, gives them an importance and influence which might otherwise be absent. Experience shows that amateurs of either sex are out of place in a great hospital. If women were to be appointed on the committees they might naturally wish to mix themselves up with the internal working of the hospital, a line of conduct which would unfit them for the position. No great hospital can be properly administered where there is constant friction, and this danger is avoided, at present, by excluding women from the committees of management.

In many of the smaller hospitals, especially in cottage hospitals, ladies often take part in the management. In those cases where there is one woman on the committee who can control her sisters, the system has sometimes worked satisfactorily, but in practice the tendency which women have to interfere with executive details, and to take sides, has often led to a breakdown in the smaller hospitals. We were recently consulted in the case of a cottage hospital where the ladies' committee constantly interfered with the matron, and, from an absence of knowledge, showed a disposition to support the least worthy members of the nursing staff, and so to set aside the matron's authority. Here there was no single woman who had sufficient influence to keep her sisters in order, and the smooth working of the hospital was seriously interfered with. Peace was ultimately secured by the enactment of some drastic rules, which placed the matron in control, and secured her the necessary support. They provided that no interference should take place except by a resolution passed by a majority of the general committee which consisted largely of men.

Looked at from every point of view, and judged in the light of experience, it follows that the well-being of the hospitals and all connected with them has necessitated the exclusion of women, as a rule, from the committees of management. There are, however, two ways in which women may become associated with the work of hospitals with advantage to all concerned. Some hospitals, like the Great Northern Central Hospital, have a ladies' association, which interests itself in the raising of money and the provision of comforts for the poorer patients on their discharge from the hospital. Such associations have done a great deal of good, and in this direction we should welcome the formation of such an association, in connection with every hospital of importance throughout the country. Again, in hospitals for incurables, where the patients, when once admitted, spend the rest of their lives in the institution, women ought to be admitted to the committees of management. Some years ago, the necessity for the presence of women on these committees was proved, at an inquiry held, under the presidency of Lord Aberdeen, into the management of the Royal Hospital for Incurables at Putney.

The longer a patient, and especially a woman patient, resides in a hospital for incurables, the more dependent she becomes on outside sympathy and friendship. There are a multitude of small matters which make for the comfort of incurables, in the case of women especially, which a man cannot fathom or properly provide for. For this reason we should be indisposed to support any hospital for incurables where the management does not provide for the admission of women to its committees. If Lady Shrewsbury, Miss Hill, and others who think with them would combine to bring about a reform in this matter, so far as hospitals for incurables are concerned, they would render a great service to the patients and the public, and do an immense amount of good.

### Lectures Upon the Nursing of Mental Diseases.

By Robert Jones, M.D.Lond., B.S., F.R.C.S.Eng., M.R.C.P.Lond., Resident Physician and Superintendent of the London County Asylum, Claybury.

### LECTURE XXII.

The insane are subject to the same fevers as are the sane; possibly their risk, at any rate among the poorer classes, in regard to one form, viz., enteric or typhoid, is greater than among the general community, as their lives are spent in institutions where large numbers congregate, none of whom can ever be bacteriologically or chemically clean. Although great care is taken in regard to the ventilation, heating, and drainage of these institutions, yet in some of them these conditions are not brought up to modern requirements, and owing to this the lives of some of the patients and staff may be endangered and even sacrificed. In support of this statement one has only to read the annual reports of the different asylums and the report of the Lunacy Commissioners to the Lord Chancellor.

Of all the means to aid a diagnosis among the insane the thermometer is the most valuable, and the nurse must know not only how to use it but also how to record the result. Before taking the temperature the index should be shaken down to a point two or three degrees below the normal, and care should be taken when holding it and shaking it with the bulb down that the index is not forced into the bulb of mercury. When placed in the mouth or the rectum—the usual places selected—it should remain from three to five minutes, and if the temperature is raised there may be fever. By the term "fever" we mean an elevation of the temperature above 101°. Anything between 103° and 105° is considered to be moderately high fever, and over 106°—hyperpyrexia—to be exceedingly dangerous.

Most morbid states in the insane, as in the sane, are, with few exceptions, accompanied by fever, and with the rise of temperature there is also a quickening of the pulse and respirations. When the fever keeps high for some time without dropping it is called *continuous* fever. If it fluctuates daily, being high at night and normal in the morning, it is *intermittent*, as is the case with hectic fever, which is well known by the pink flush on the cheeks, a pale face and free perspiration indicating phthisis, suppuration, or the formation of an abscess. If a high feverish temperature does not fall to normal, but remains high for some time, falling for a while—but not to normal—and rising again, it is called a *remittent* fever. Fever indicates many disorders, but some alterations of temperature are *typical* and of diagnostic value. A sudden rise with a chill and rigor indicates pneumonia. A gradually increasing and continued daily temperature may indicate typhoid. A rise at night and a fall in the morning indicates phthisis, or a rise of temperature may indicate one of the so-called infectious fevers which are often called *specific* or *zymotic*, in the belief that they are caused by certain specific germs or organisms. The spread of these fevers in an institution is a matter of grave concern to the health and possibly to the life of the inmates, and it is also a serious expense to the responsible authorities. In cases of specific fevers the particles of infection float in the air, or they are conveyed by the clothing, get into the food, or are communicated by the breath from one person to another. It is important, therefore, that the cases should be recognised early so that proper means may be taken for their isolation, disinfection and treatment. The *infectious* fevers mostly prevalent—*contagious* is a term now rarely used, as the word infectious covers contact and other modes of communication—are usually described as chicken-pox, scarlatina, small-pox, measles, typhus, typhoid, diphtheria, mumps, and cholera as opposed to non-infectious fevers, such as rheumatic fever, ague, and malaria. Consumption, due to a communicable micro-organism is not regarded as a fever, but classified like other diseases, such as syphilis, etc., of similar microbic origin. In ordinary text-books on nursing, the infectious fevers have no place and are treated apart; but as the mental nurse may be called upon to look after any mental case with fever, we shall allude briefly to the danger of communication and the means of identifying fevers, with short directions referring to precautions against their spreading. All have in common a *period of incubation*, a time after the entrance of infection into the blood during which the fever develops, but without any definite outward symptoms. When this stage is over and signs of the fever begin to appear this is called the *period of invasion*, and it is during this stage that a feeling of fatigue, headache, dulness, or a rigor may make their appearance, the temperature rises, the pulse quickens, and other symptoms of feverishness follow, together with the *rash*, which is the obvious and more diagnostic symptom, a stage which is now called the *period of eruption*, during which, for each specific fever, various skin affections appear which characterise the disease. In chicken-pox a crop of vesicles appears within 24 hours of the symptoms; in small-pox hard small spots appear on the third day of the symptoms, which spots soon become vesicles, and finally contain pus, and then crust, leaving a scar when the crust falls.

Headache, vomiting, and pain in the loins are the special symptoms characterising the "invasion" period of small-pox. In scarlet fever there is a general rosy rash appearing on the second day of invasion and there is often much sore throat. In measles small red papules in groups are seen on the fourth day of the illness near the hair-line on the forehead, and there is often much watery mucus at the eyes and nose. In typhus—which is now rarely met with—a dull red rash appears on the sixth day after symptoms of serious and intense illness. In typhoid fever (which is apt to occur in the autumn), after the first week of ill-defined illness, small red spots appear daily on the abdomen, which gradually fade, being replaced by fresh ones, which again disappear, in separate crops. There is also marked mental dulness and usually diarrhœa.

The danger of infection in these fevers is from the breath in measles, chicken-pox and small-pox, scarlet and typhus fevers; from the skin in chicken-pox and small-pox and scarlet fever; from the stools and the urine in typhoid, and from the emanations or exhalations of the body and clothing generally in all these except typhoid. In typhoid fever if the stools have soiled the clothing or bedding there is a risk of infection from them. Death takes place from bronchitis in measles, from blood-poisoning and dropsy in scarlet fever, blood-poisoning in small-pox, and from perforation of the bowels, hæmorrhage, or collapse in typhoid.

The precautions to be taken in nursing the insane under fevers are immediate isolation—in fact, this is the common treatment for all infections. Every patient and every other person who has been in contact with a small-pox case should be re-vaccinated and the nurse must see that every rag used for a scarlet fever or a measles case is burnt, also to see that the stools and the urine of a typhoid fever case are burnt or disinfected before putting down the drains. In addition, for scarlet fever the throat is washed with some medicated application such as chlorine water, or inhalations are used. During convalescence care must be taken to prevent the scales of skin which are shed from dispersing into the air, by the use of warm baths and the application of carbolic oil or camphorated oil, also to avoid chills and the great dangers from kidney affections. In small-pox the patient must be kept clean and especial care should be taken to keep discharges from entering the eye. In measles all complaints about the ears should be noticed and discharges treated with care. In typhoid, which of all fevers, for recovery depends most upon good nursing, the patient will be in bed for weeks and during this time the body should be kept clean, the hair kept short and the feeding most carefully attended to. Rest in bed must be rigidly observed as the best precaution against ulceration and perforation of the bowels, the diet should be milk and, possibly, nothing else. Nutrient enemata with stimulants are given in cases of extreme exhaustion. As to diphtheria, the patient is isolated and the throat painted or swabbed with antiseptics as may be ordered. Constant nourishment helps to maintain the strength and a careful watch must be kept for laryngeal symptoms. During convalescence much care is necessary, as there is a liability to fainting and paralysis. The recovery from diphtheria is often a matter of months. In nursing cases of erysipelas similar precautions against the spreading should be taken by isolation, and the room must be carefully disinfected by burning sulphur or vaporising formalin after removing the patient and sealing up all exits and chinks.

The sick-room for insane persons should ordinarily be on the ground floor, but for infectious cases the top floor is best suited. In the case of an insane person suffering from infectious disease, *i.e.* when both conditions are combined, it is best to isolate on a well-ventilated ground-floor flat. This should be stripped of all unnecessary accessories—curtains, pictures, and furniture. No other person should be permitted to share this room, and the patient must be under constant observation night and day. The usual sheet steeped in carbolic (1—40) should be hung over the door of the room, and all body and bed-linen should be taken outside and soaked for an hour or more in disinfectants before being taken for further disinfection into the special chamber for the purpose, such as the Washington Lyons apparatus. Nurses in attendance must observe strict rules as to disinfection and change, and everything used as an accessory must be freely disinfected.

*(To be continued.)*

### How to Strap a Leg for the Cure of an Ulcer.

### EXAMINATION QUESTIONS FOR NURSES.

The question was as follows:—If requested by a doctor to strap a leg for the cure of an ulcer half way up the shin, how would you proceed? Describe your preparation of the limb—of the strapping—and of your application of the strapping to the leg. In what manner would you remove the plaster when it required removing?

### First Prize.

When applying strapping to an ulcer, first wash the leg with water and an antiseptic soap and shave off any hair that is on the area to be strapped, to render less painful on removal of strapping. Wash the wound with an antiseptic solution, and if a dressing has been ordered spread the ointment on the smooth side of the lint and cut to exact size of the ulcer. Cut the strapping into strips about one-and-a-half inch in width, each piece being long enough to pass once and a half round the limb.

Start just above the ankle and place the centre of the strip of strapping at the back of leg and cross in front in figure of eight style. Work upwards, each piece of strapping overlapping the other nearly half an inch.

Should there be much discharge small holes may be cut in the strapping near the seat of wound to allow the discharge to escape. Carry the strapping up to just below the knee. Cover the limb with a layer of antiseptic wool and bandage firmly from below the ankle to just below the knee.

To take off the strapping loosen at the lower edge and cut up the side of the leg with blunt pointed scissors, when it usually peels off in one piece with very little trouble. If there is much difficulty in removing it, turn back the piece that is loose and apply a swab of wool soaked in turpentine to the part that is fast and pull gently (taking care that the turpentine does not touch the ulcer). Clean the remains of the strapping off the leg with turpentine and wash after with soap and water. "Yuntha."

### Second Prize.

Before I begin to apply the strapping I should thoroughly cleanse the leg with soap and water, next thoroughly wash the ulcer with a lotion (generally carbolic 1-40). I should inquire from the doctor what he would prefer applied to the wound before the strapping (probably he would order cyanide gauze), if so, I should next apply a few layers. I should then proceed with the plaster from about two inches below the wound to about two inches above. Each piece of plaster requires to be one and a half inch wide and long enough to go once and a half round the leg, crossing in front, the ends passing upwards on either side, each strip overlapping each other by one third, keeping it perfectly smooth and free from creases. Care must be taken to gently draw the edges of the wound together to prevent stretching, but too great traction must not be made in case of œdema, when patient would not be able to bear the pressure. Before cutting the strapping into strips I should wipe it with a clean cloth to remove all dust and loose particles of plaster. It should either be warmed in front of a fire or round a tin containing boiling water, the linen side next to the heat. I should not damp with turpentine, because it might cause irritation of the skin.

To remove I should pass a director underneath the plaster (being particular not to go over the wound) followed by the scissors so as to remove the whole at once, as it is more comfortable for the patient than tearing each piece off separately. After the removal I should take the marks of the strapping off with turpentine and finish with soap and water. Cleaning the ulcer with carbolic 1-40 if it should not be quite healed. "Punch."

### The Prize Winners.

On the whole the papers this month are good. "Yuntha" is successful in gaining the first prize because she attends to three important points—the necessity of strapping the entire space between the ankle and knee; the advisability of leaving small holes in the strapping in the region of the ulcer for the escape of discharge; and because she sees the necessity of removing the plaster in one piece. It is better however, to cut it up at the back instead of the side, to pull on both edges at once towards the ulcer. Her plan of using a swab of wool soaked in turpentine is bad—there is danger of injury to tender skin, somewhat soddened by the strapping. With a firm but gentle touch, without hesitation and uncertainty, it is easy to take off the whole in one piece. It very seldom sticks over the ulcer itself, the discharge tending to soften and even decompose the strapping. "Punch," the second prize winner, also speaks of cleaning off strapping marks with turpentine. If anything beyond soap and water be needed, olive oil is the best thing to use. Her paper is very good, but she speaks only of applying strapping from two inches below to two inches above the wound. It should cover the leg from ankle to knee, and if circumstances permit, it is much better to begin from the toes, so as to secure even support and pressure and avoid any tendency to swelling of the foot. In some cases, where the circulatory system is deranged, this mode of proceeding is absolutely necessary.

### Honourable Mention.

This is gained by "A Climber," "Maidenhair," "Central," and "Katrina." "A Climber's" paper is admirable, but unfortunately she writes carelessly; she says she should strap from the toes to the knee, and only provides herself with "nine or more strips of strapping," whereas at least two dozen would be needed for a full-sized leg. "Katrina" would find strapping 1 inch wide too narrow.

### Question for December.

How would you carry out an order to sponge a fever patient with tepid water? (2) How would you proceed to cold-sponge a similar case?

### Rules.

The competition is open to all. Answers must not exceed 500 words, and must be written on one side of the paper only, without divisions, head lines, or marginal notes. The pseudonym, as well as the proper name and address, must be written on the *same* paper, and *not on a separate sheet.* Papers may be sent in for fifteen days only from the day of the publication of the question. All illustrations strictly prohibited. Failure to comply with these rules will disqualify the candidate for competition. Prizes will be awarded for the best two answers. Papers to be sent to "The Editor," with "Examination" written on the left-hand corner of the envelope.

In addition to two prizes honourable mention cards will be awarded to those who have sent in exceptionally good papers.

N.B.—The decision of the Examiner is final, and no correspondence on the subject can be entertained.

Any competitor having gained three prizes within the current year shall be disqualified from taking another until 12 months shall have expired since the first prize was gained.

### Nurses' Pensions at the Middlesex Hospital.

A proposal, signed by nearly a dozen members of the weekly Board, with Lord Cheylesmore's name at the head, was before the Quarterly Court of the Middlesex Hospital on Thursday, under the chairmanship of Prince Francis of Teck, with reference to an increase in the pensions to sisters and nurses. As the law of the hospital stands, the Board has power, if it see fit, and subject to the approval of the Court, to reward the long and faithful service of any salaried officer, including sisters and nurses, by a pension not exceeding, after 20 years, two-thirds of the salary, or if disabled for duty by sickness or accident incurred whilst on duty in the service of the hospital after not less than ten years, one-half of the salary. The proposal was to increase the pensions of sisters and nurses, in special cases, by a sum not exceeding £2 per head.

Mr. Pearce Gould rose to bring forward an amendment. Having explained that he did not wish to run counter to the resolution, but rather to amplify it, since the Governors would, he was sure, welcome any proposal to recognise the services of the nursing staff, he drew attention to two pensions of £26 13s. 4d. and £18 13s. 4d. respectively, after 23 years' service (in the one case as sister, and the other as nurse), which the Board had just sanctioned by passing the quarterly report. The pensions equalled two-thirds of the salaries received. At first sight, he continued, a pension of two-thirds of the salary might appear a very liberal one indeed. But the money paid in salary was only a small part of the pay received by the nurses in question; board, lodging, and grants for uniform amounting to £24 a year, reduced the pension to something much below one-half of the emoluments received during active service; and to give on retirement only two-thirds of the actual money payment was in no way equivalent to the pension to which a resident clerk or servant of the hospital was entitled on retirement. When the figures were worked out, on the basis of the nurse's pension just granted, the pension of £18 13s. 4d. was less than 7s. 6d. a week, viz. about one shilling a day. He happened to know that particular nurse, and the circumstances under which she was retiring, invalided, and, so far as could be seen, unable to pursue her calling as a nurse, or to undertake any labour of a severe kind; indeed, he was very doubtful whether she could by her efforts add to this small pittance of a shilling a day. He was quite sure that the Governors of the hospital would view with great regret the compulsory resignation of a nurse whose services for 20 or more years were rewarded by her being turned adrift with such a pittance. He was well aware that the whole question of pensions was beset with very great difficulty. There was first of all the great variety in the circumstances of nurses who left the hospital; a great number married, others had homes to which they were able to return, but there remained a large proportion who after a long period of service had nothing to fall back upon but a pension, and possibly a small amount of savings (and on a salary of only £24 a year it was not reasonable to suppose that a nurse could add any notable sum to her pension). After remarking on the possibility of money presents in certain kinds of nursing work, Mr. Gould said he thought it exceedingly wise that the Board should be asked to be given discretion in the amount of the pension, and he hoped the Governors would enlarge their discretionary powers, not merely to the extent of an additional £2, but, if the Board saw fit, up to the full amount of the salary being paid to the sister or nurse at the time of her leaving the hospital after 20 years' service, and in special cases.

Col. Needham seconded the amendment, and it was passed unanimously without further remark.

A vote of thanks to the Chairman closed the proceedings.

### TRAVEL NOTES AND QUERIES.

By our Travel Correspondent.

Pensions in Mentone (Lonsdale).—Hôtel and Pension Littoral charge as their lowest terms 8 frs. per day. Hôtel and Pension Santa Maria from 7½ frs.—this is very good. Hôtel Beau Rivage from 8 frs. Hôtel Malta from 7 frs. Hôtel Turin from 8 frs. Are you aware that the difference in terms is always carried out in the position of your rooms, not in the quality or quantity of the meals. Pension terms from 7 frs. and 8 frs. in a good house always means a room with a north aspect. If you are healthy and much out of doors this is of little consequence, but for one much confined to the house a southern aspect is of the first importance and it is a luxury for which you must pay dearly on the Riviera. I should advise you to spend the first part of your time at Mentone and then to go on to Bordighera, Rapallo, or Pegli.

Rules in Regard to Correspondence for this Section.—All questioners must use a pseudonym for publication, but the communication must also bear the writer's own name and address as well, which will be regarded as confidential. All such communications to be addressed "Travel Correspondent, 'Nursing Section of *The Hospital*,' 28 Southampton Street, Strand." No charge will be made for inserting and answering questions in the inquiry column, and all will be answered in rotation as space permits. If an answer by letter is required, a stamped and addressed envelope must be enclosed, together with 2s. 6d., which fee will be devoted to the objects of "The Hospital" Convalescent Fund. Ten days must be allowed before an answer can be published.

### The Central Midwives Board.

### THE CHAIRMAN'S CORRESPONDENTS.

A MEETING of the Central Midwives Board was held at the offices, 6 Suffolk Street, on Thursday last week. There were present: Dr. Champneys (Chairman), Dr. Cullingworth, Mr. Ward Cousins, Miss Rosalind Paget, Sir William Sinclair, Miss Wilson, and Mr. Parker Young.

A letter was read from Mrs. Heywood Johnstone, thanking the Board for their resolution of sympathy passed at the meeting of October 27th. Miss Oldham wrote resigning her position on the Board owing to absence abroad, and the secretary was instructed to express the Board's regret, and to communicate with the Royal British Nurses' Association, whose representative she was. The honorary secretary of the Metropolitan Counties Branch of the British Medical Association wrote, enclosing copy of a letter addressed by the branch to the London County Council, with a pamphlet, asking for the Board's co-operation with the Council in obtaining powers from Parliament to pay registered medical practitioners when called in by midwives in emergencies. Mr. Duncan had in reply pointed out that the Board had already approached the Privy Council on the subject some months ago and advised the Honorary Secretary to take steps to bring the question before that body. The Board being entirely in sympathy with the wishes of the Association, it was proposed by Mr. Parker Young, seconded by Mr. Ward Cousins, and carried unanimously, that a letter be sent again drawing attention to the action already taken, and expressing willingness to co-operate by any means in their power.

A letter from the Superintendent of St. Clement's Maternity Home, Fulham, was considered, explaining certain facts with regard to which a letter had been addressed to her by the board. It was agreed to take no further action.

Some discussion occurred over a letter from the clerk of the Monmouthshire County Council, asking the Board's construction of the words "otherwise than under the direction of a qualified medical practitioner." Sir William Sinclair considered that, in reply, the clerk should be simply referred to the words of the Act. Miss Wilson objected to the absurdity of answering an inquirer in his own words, pointing out that it was the board's "construction" of them that was asked for. Ultimately, it was agreed that a letter should be written to the effect that the point involved was a legal one upon which no authoritative decision had yet been given.

The Chairman then intimated that he had personally received two or three letters which he wished to read to the Board. One from Dr. Dakin, with reference to the proposed date of the first examination, was postponed till the next meeting for discussion. Two others, one from Mrs. Wallace Bruce, chairman of executive committee of the Association for Promoting the Training and Supply of Midwives, and another with reference to the Birmingham Lying-in Charity, dealt with the same subject, namely, the annoyance felt at the difficulty experienced in obtaining from the Board consideration of the difficulties of newly-formed institutions, anxious to comply with the requirements of the Act, but hindered in so doing, as they allege, by the "unreasonableness" of the Board, which declined to advise or explain what was expected of them. It was finally suggested by Miss Wilson, after a discussion in the course of which Sir W. Sinclair said he failed to "understand" the letters, and "moved the previous question," that the letters should be brought forward at the next meeting. Miss Paget supported the resolution, which was carried by the chairman's casting vote, Sir W. Sinclair, Mr. W. Cousins, and Mr. Parker Young, voting against.

The financial statement was passed, and a number of applications from institutions, teachers and midwives postponed to an adjourned meeting on December 1st.

Miss Wilson then introduced the following resolution:—

That in order to carry out the resolution of the board, on April 28th, it be resolved that the board take steps forthwith for the appointment of a trained woman inspector.

She explained her reasons for thinking it desirable that such an inspector should be appointed, to visit such institutions as might be considered desirable by the Board, when fuller information was required of the management and working of those applying for recognition. Sir William Sinclair and Mr. Parker Young opposed, the former again "moving the previous question," and after a prolonged discussion the motion was carried, with the omission of the words "trained woman," and the addition of the following, "Willing to act under the conditions specified under that resolution."

The following resolution, moved by Dr. Cullingworth and seconded by Miss Paget, was after considerable discussion, postponed.

That during the consideration of matters having reference to the judicial or penal powers of the board, or of applications for recognition or approval (under Section C. of the rules) on the part of institutions (as training schools) of medical practitioners (as teachers) or of midwives (entitling them to sign Forms III. and IV. under Rule C. 1 (2)) the representatives of the Press shall be requested to withdraw.

Sir William Sinclair strongly objected, stating that he had complete confidence in the discrimination of the Press. Throughout a long experience he could remember no instance of indiscretion on their part. Dr. Cullingworth said he wished in no way to reflect upon the Press; he fully trusted their discretion, but having regard to the delicacy of the matters which came before the board he thought there were times when discussion should be held with closed doors. It was ultimately decided to ascertain the procedure of the General Medical Council on similar occasions, and to postpone the consideration of the resolution meanwhile. Discussion as to the admission of the Press at the adjourned meeting when a penal case would be before the board, terminated in a decision to admit them.

A resolution was moved by Miss Paget, seconded by Dr. Cullingworth and carried, embodying certain principles to be followed in approving midwives for signing forms III and IV.

(*a*) Midwives who are employed by an institution or association with a responsible committee shall, if otherwise suitable, be approved, but they must in all cases provide a recommendation from a responsible official of the institution.

(*b*) Midwives who have held positions such as the above, but who are now working independently, shall state the names of such institutions or associations, and the dates when employed by them.

(*c*) The cases of midwives who do not fulfil either of the above requirements shall be judged on their own individual merits, consideration being given to the requirements of their districts.

### Novelties for Nurses.

BY OUR SHOPPING CORRESPONDENT.

### CHRISTMAS CARDS.

I HAVE received from the Scientific Press specimens of Personal Christmas Cards, which they are offering from 2s. per dozen. Purchasers may supply their own wording. The selection is on view at 28 Southampton Street, Strand. The cards are of good quality, and the designs simple and pretty.

### Appointments.

Alexandra Hospital for Children with Hip Disease, Queen Square, London.—Miss E. Margaret Fitch has been appointed matron. She was trained at the Royal Isle of Wight Infirmary, Ryde, and has since been staff nurse at the London Temperance Hospital, charge nurse at the Brook Hospital, Shooters' Hill, sister at the National Hospital, Queen Square, London, night superintendent at the City of London Hospital for Diseases of the Chest, Victoria Park, and assistant matron of the Children's Hospital, Paddington Green, W.

Central London Ophthalmic Hospital.—Miss A. L. Harris has been appointed charge nurse. She was trained at the Middlesex Hospital, and at Queen Charlotte's Hospital, London. She has since been staff nurse at Addenbrooke's Hospital, Cambridge.

Children's Hospital, Nottingham.—Miss Mapson has been appointed staff uurse. She was trained at the Victoria Hospital for Children, Chelsea, and has since been staff nurse at the Cottage Hospital, East Cowes, Isle of Wight.

City of London Hospital for Diseases of the Chest, Victoria Park, E.—Miss Edith M. Chapman has been appointed house sister. She was trained at Addenbrooke's Hospital, Cambridge, and afterwards became night sister at Ryde Hospital, Isle of Wight. She has since been ward sister at the Norfolk and Norwich Hospital, and has also done private nursing.

Croydon Union Infirmary.—Miss E. M. Northover has been appointed night superintendent, and Miss Gertrude Mary Evans, ward sister. Miss Northover was trained at the Middlesex Hospital, London, where she was afterwards staff nurse. She has since been theatre and ward sister at Bethnal Green Infirmary. Miss Evans was trained at St. George's Infirmary, Fulham Road, London, where she has since been sister. She has also been night superintendent at Fulham Infirmary, Hammersmith, and temporary nurse at the Norfolk and Norwich Hospital.

Gloucester General Infirmary.—Miss Marian Mann has been appointed assistant matron. She was trained at the Preston Royal Infirmary, and has since been charge nurse at the Park Hospital, Lewisham, night sister at the Oldham General Infirmary, and sister at St. Mary (Islington) Infirmary, Highgate Hill, London.

Inwood's Cottage Hospital, Alton, Hants.—Miss E. H. Phillips has been appointed matron. She was trained at the Royal Hospital, Portsmouth, and has since been sister. She has also been charge sister and night superintendent at the Royal National Hospital, Ventnor.

Ledbury Cottage Hospital.—Miss Alice Lee Smith has been appointed matron. She was trained at the Royal South Hants and Southampton Hospital, has been charge nurse at Dover General Hospital, sister at Croydon General Hospital, and sister at the Royal South Hants Hospital. She also served for two years and a half in South Africa as a member of the Army Nursing Service, Reserve, obtaining both war medals.

Open-Air Sanatorium for Consumption, Limpley, Stoke.—Miss Jessie Creighton has been appointed matron. She was trained at the London Hospital, Whitechapel, E., and has since been night superintendent at Bristol Royal Infirmary.

Rest for the Dying, Dublin.—Miss G. H. Maguire has been appointed charge nurse. She was trained at the Adelaide Hospital, Dublin, and was for some time on the private staff. She has also done private nursing.

West London Hospital, Hammersmith.—Miss S. A. Musson has been appointed assistant matron. She was trained at Liverpool Royal Infirmary, and has since been sister and night superintendent at Hull Royal Infirmary, sister of typhoid wards at Hull Sanatorium, and night superintendent at the Royal Hospital for Diseases of the Chest, City Road, London.

Whitehaven and West Cumberland Infirmary.—Miss Lillie Evans has been appointed matron. She was trained at the Royal Infirmary, Newcastle-on-Tyne and has since been assistant matron at the Stanley Hospital, Liverpool.

### Everybody's Opinion.

### THE NURSES' LIMIT.

Mr. Conrad W. Thies, secretary to the Royal Free Hospital, writes to us: My attention has been called to an article in the last issue of The Hospital, *Nursing Section*, under the title of "The Nurses' Limit," which was evidently inspired by a very inaccurate and misleading paragraph which appeared in one of the London daily newspapers. The facts of the case are as follows:—The patient referred to walked into the hospital with another youth and stated that he had met with a slight accident, of which he made light. He was seen by one of the *student dressers* on duty in the casualty receiving room and was not seen by a nurse at all, the nurse on duty in the casualty room being absent at the time. The wound was washed and a temporary dressing was placed thereon by the student dresser, the patient being left on a couch until he could be seen by the casualty house surgeon, who had been temporarily called away. When that officer returned, in about a quarter of an hour, it was found that the patient had disappeared without the knowledge of the dresser, who was busy with other patients. The father of the patient called the same evening and reported that his son had been sick; he was asked to bring his son to the hospital at once and a bed would be provided, but he did not do so, and nothing further was heard of the case until one of our resident medical officers was summoned to the inquest. It is much to be regretted that the writer of the article in your paper did not take the trouble to ascertain the actual facts before commenting on a newspaper report, which reports, as you are well aware, are frequently very unreliable. An inquiry addressed to the hospital would at once have elicited the facts of the case. I may state that it is a strict rule of this institution that no patient is to be allowed to leave the hospital without having been seen by a qualified medical officer, and this rule applies to every case however slight. I would ask you to give these facts publicity in your next issue, as the comments in the article referred to are calculated to give a very unfavourable impression as to the manner in which the work of this hospital is conducted.

[The report referred to was not contradicted by the Royal Free Hospital authorities, and the fair presumption was, therefore, that the statements were in accordance with the facts. In the ordinary course it is our custom to make an independent investigation when the facts are in dispute.—Ed. The Hospital.]

### Presentations.

Royal Halifax Infirmary.—Miss James, on resigning her post as matron of the Royal Halifax Infirmary, was the recipient of some very handsome gifts. The nursing staff presented her with a solid silver cake basket, the house-surgeons with a massive oak and silver tray, the servants with a silver triple dish and silver flower pot, and the porters with a bronze statue holding a rose bowl. She also received presents from past and present patients and personal friends.

### Where to Go.

Exhibition of Old Embroideries and Antique Laces.—Messrs. Debenham and Freebody, Wigmore Street, Cavendish Square, W. Admission free.

### Echoes from the Outside World.

### Movements of Royalty.

The King left London on Monday afternoon and travelled in a dense fog to Wolferton, the train, however, arriving at the station three minutes before the scheduled time. His Majesty proceeded in a closed motor-car to Sandringham, where the Queen and Princess Victoria awaited his arrival. On Tuesday the King and the Prince of Wales had some rabbit-shooting, the Queen and the Princess of Wales joining them at lunch. On Thursday, the Queen's Birthday was celebrated in the usual manner. Salutes were fired off by the Army and Navy in honour of the anniversary. Princess Louise, Duchess of Argyll, who visited Glasgow on Tuesday in order to attend a meeting in connection with the Maternity Hospital, received on the same day, at the University of Glasgow, the honorary degree of Doctor of Laws.

### The King and Queen of Portugal.

The King and Queen of Portugal concluded their stay at Chatsworth on Friday. An amusing feature of the visit was a lively snowball fight in which the King took part. In London on Saturday the King, attended only by an equerry, made several purchases in the Bond Street shops, and, not being recognised in some cases, carried his small parcels away in his pockets. The King and Queen left Buckingham Palace for Wood Norton on Saturday, on a visit to the Duke and Duchess of Orleans. Their Majesties were met at the station by the Duke of Orleans in a cream-coloured motor-car. The avenue was decorated with red, white, and blue lights, but they were barely visible in the murky atmosphere. On Sunday the royal guests early attended mass, and after luncheon were photographed in the garden previous to going for a long walk through the park. In the evening a great display of fireworks quite illuminated the snow-clad landscape, the trees on the lawn being hung with chains of electric light. On Monday and Tuesday the day was devoted to sport, and on Wednesday the King and Queen returned to Buckingham Palace.

### A Grand Duke's Will.

The Grand Duke of Mecklenburg's will—which was proved at £83,000 personal estate—contains some touching allusions to his wife. He says:—" We bequeath to our dear Consort and Love, the Grand Duchess, whom we at this stage once more thank with all our heart for the love and fidelity that she has at all times shown, and also for her invariable affection, as the share of the estate due to her by virtue of her appointment as heiress, . . . an annuity for life of 60,000 marks." The will also provides for the residence of the Grand Duchess in the Castle, for the use by her of "the little kitchen," for the feeding and keeping of her ponies, the cleaning and housing of her pony carriage, and the provision for her use of seven carriage horses, a town carriage, a brougham, and "a nice open carriage, a coachman, an outrider, and a side rider."

### Lord Curzon's Return to India.

The departure of Lord Curzon of Kedleston to India, in order to resume, for a further period, the duties of Viceroy, took place on Thursday last week, a little more than six months since he came to England for a holiday. As every-one knows, his leave has been prolonged in consequence of the serious illness of his wife, who was removed a few days before her husband quitted England to Highcliffe Castle, Christchurch, where she will remain for some time. She continues to make satisfactory progress towards recovery, but was, of course, unable to see Lord Curzon off at Charing Cross. Several of the Viceroy's near relatives and friends were present to bid him farewell, and on his arrival at Dover, later in the day, he referred to his wife in hopeful terms.

### England and Russia.

The Convention between England and Russia was signed on Friday at St. Petersburg, and the text of it was published on Monday. There are eight articles, of which the one relating to the scope of the International Commission of Inquiry is to the effect that "the Commission shall inquire into and report on all the circumstances relative to the North Sea incident, and particularly on the question as to where responsibility lies, and the degree of blame attaching to the subjects of the two high contracting Powers or to the subjects of other countries in the event of their responsibility being established by the inquiry."

### The War in the Far East.

The Premier of Japan, on the eve of the assembling of the Diet, made a very important declaration, in the course of which he said: "Russia must see that the war cannot be concluded by the issues of a few battles. With us the war means life and death, and no one of our 45,000,000 of brethren remains ignorant of the vital issue at stake. We are prepared to sacrifice the last man and the last yen in the war." The Premier, observing that everything seems to hinge on the fall of Port Arthur, adds: "But I do not console myself with the thought that the capture of this ill-fated fortress will bring the war to a speedy termination."

### Destruction of Historic Mansions.

In a week no fewer than three historic mansions have been either entirely, or partially, destroyed by fire. In the small hours of Thursday morning last week Westbury House, near Petersfield, Hampshire, the seat of Colonel Le Roy Lewis, was the scene of a terrible conflagration, and but for the bravery of the gallant owner, several lives would have been lost. As it was, one woman died as the result of her injuries. The older portion of the mansion, which was erected in the time of Queen Anne, is in ruins, and only the small new wing remains, On the following morning Enville Hall, Staffordshire, one of the three seats of Catherine Countess of Stamford, was wrecked, the fire breaking out at one o'clock and lasting until mid-day. The original mansion was erected in the reign of Henry VIII., but the present hall was built in the middle of the eighteenth century. The extensive picture gallery contained family portraits and paintings by several great masters, and among the heirlooms were gold vases won by famous horses bred at Enville. The pecuniary damage is estimated at tens of thousands of pounds. Stanwell Place, Staines, was also partly destroyed by fire last week. This commodious mansion formerly belonged to the Windsor family, but it passed into the hands of Lord Knyvet in the time of James I., and had several other owners before Lord Dunmore purchased it in 1720. Soon after his death in 1752 it was purchased by Sir John Gibbons, and has since remained in the possession of his descendants.

### The Case of Mr. Adolf Beck.

The Committee appointed to inquire into the case of Mr. Adolf Beck, who suffered a long term of imprisonment in mistake for another man, named John Smith, have issued their report. At the outset the Committee, who consisted of the Master of the Rolls, Sir John Edge, and Sir Spencer Walpole, record their opinion that there was "no shadow of foundation for any of the charges made against Mr. Beck, nor any reason for supposing that he had any connection with them." The police are exonerated from blame, but the judge who tried the case at the Old Bailey is blamed for not fully appreciating the grounds of the defence, and the report renders it clear that the system under which such gross injustice was possible is in urgent need of reform

### Notes and Queries.

**FOR REGULATIONS SEE PAGE 12.**

*Spinal Carriage.*

(60) Can anyone tell me where a spinal carriage could be hired, and the cost? It is for a nurse recovering from a long and expensive illness.—*Katherine T.*

Messrs. Carter, 6A New Cavendish Street, Portland Place, would probably supply one at sufficiently moderate cost; or advertise stating case.

*School Nurses.*

(61) Can you favour me with details of the work done by nurses under the Education Committee of the London County Council in the elementary schools?—*E. C.*

See article in the Nursing Section of THE HOSPITAL for July 30th entitled "The School Nurse Established," p. 254.

*Patent.*

(62) Will you tell me how I could get an invention patented? It is a specimen cover for urine glasses.—*M. F.*

Write for particulars to the Patent Office, 25 Southampton Buildings, W.C.

*Care of Baby.*

I have charge of a delicate baby going out to India, who occasionally requires white of egg. Will you kindly tell me if it would be possible to take some eggs on the voyage by packing them in a sealed bottle?—*F. H.*

Eggs are kept in the cool chambers of the ship. Write and ask the authorities of the vessel about it.

*Chiropody.*

(63) Will you kindly tell me how I can be trained in chiropody?—*Memo.*

Only by making arrangements for private tuition with a practical chiropodist.

*Superfluous Hair.*

(66) Will you kindly tell me if it is possible for superfluous hair to be removed? I have had hair removed myself by the people who advertise to do it by electrolysis, but the operation seems only to stimulate the growth. I should be so thankful if you could tell me of a skilful doctor and if there is a sure cure.—*A. E. S.*

Electrolysis is a sure cure in skilful hands. Doctors who make a special study of the hair are attached to the different skin hospitals in London.

*Epileptics.*

(67) I should be extremely obliged if you will kindly inform me if there are any "homes" for epileptic patients in England?—*A. J. G.*

There are the homes of the National Society for Promoting the Employment of Epileptics at Chalfont St. Peter, Bucks (apply 12 Buckingham Street, Strand, W.C.); the Meath Home of Comfort for Female Epileptics, Westbrook, Godalming; the St. Luke's Home for Epileptic Churchwomen, 36 Parkwood Road, Bournemouth; and the Home for Epileptics, Maghull, near Liverpool (apply the Hon. Secretary, 2 Exchange Street East, Liverpool).

*Surgical Cases.*

(68) Can you tell me which of the London infirmaries receive surgical cases and accidents?—*A. P.*

Amongst others, the City of London Infirmary, Bow Road, E., and Fulham Parish Infirmary, St. Dunstan's Road, Hammersmith, W. But surgical cases and accidents are treated at all.

*Northern India.*

(69) Can you tell me where to obtain particulars as to posts for trained nurses in Northern India, Cashmere preferred?—*E. S. F.*

The Up-Country Nursing Association for Europeans in India would be the most likely to give you the information. Apply to Mrs. Sheppard, 10 Chester Place, Regent's Park, N.W.

*Hospital Training.*

(71) Will you kindly tell me where to obtain training in a children's hospital where salary is given?—*F. C. C.*

See "The Nursing Profession: How and Where to Train."

**Handbooks for Nurses.**

| | Post Free. |
|---|---|
| How to Become a Nurse: "The Nursing Profession" ... | 2s. 4d. |
| "Nurses' Pronouncing Dictionary." Terms and Treatment... ... | 2s. 0d. |
| "A Handbook for Nurses." Dr J. K. Watson ... ... | 5s. 4d. |
| "Elementary Physiology for Nurses" ... ... ... | 2s. 0d. |
| "Swedish Massage and Movements" ... ... ... | 2s. 6d. |

Of all booksellers or of The Scientific Press, Limited, 28 & 29 Southampton Street, Strand, London, W.C.

### For Reading to the Sick.

### FAITH IN GOD.

ONE adequate support
For the calamities of mortal life
Exists—one only;—an assured belief
That the procession of our fate, howe'er
Sad or disturbed, is ordered by a Being
Of infinite benevolence and power;
Whose everlasting purposes embrace
All accidents, converting them to good,
The darts of anguish fix not, where the seat
Of suffering hath been throughly fortified
By acquiescence in the Will Supreme,
For time and for Eternity; by faith,
Faith absolute in God, including hope,
And the defence that lies in boundless love
Of His perfections; with habitual dread
Of aught unworthily conceived; endured
Impatiently; ill-done or left undone,
To the dishonour of His Holy Name.
Soul of our souls! and safeguard of the world!
Sustain, Thou only canst, the sick of heart,
Restore their languid spirits, and recall
Their lost affections unto Thee and Thine!

*Wordsworth.*

Little reck they of the disturbance of time who gaze on eternity. These are they who can be practical yet contemplative, humble yet vigorous, self-forgetting yet full of resource; who can be bright yet serious, grave yet full of sunshine, large in view, yet willing and wisely loyal to the claims of life's details of duty; who do not fail in sympathy for other's trials, because they deeply feel their own responsibility; whose repentances are deeper, quicker, more lasting than their faults; who grow in the power of spiritual apprehension, and gain ground in the exercise of a disciplined life; who have "songs in the night," and sunlight on the darkest day; who draw gentleness from the springs of laughter, and sweetness from the fountain of tears. These are they for whom the grave may, indeed, have sadness, but over whom it cannot assert a sway of unconquerable gloom; for whom life is filled with ever-advancing experiences of blessedness, and for whom death the destroyer has lost the power to dismay.

. . . . . . . . .

There is only one path for all towards growth as towards perfection, and that is the path in time illuminated by a temper of eternity, and tracked out for us all by Christ.

The waves of eternity beat upon the shores of time. Cold and cruel they seem to be. Cross them with the wings of a spirit buoyed up on heavenly hope and childlike confidence, and you find there is an eternal country, which, when you must be driven from this little scene of your life's struggle, will make you an eternal home.

. . . . . . . . .

The human soul exiled from its natural home must lift its eyes above the mountains and see the morning dawn. "We are looking, not at the things which are seen, but at the things which are not seen: for the things which are seen are temporal, but the things which are not seen are eternal."

*Phillips Brookes.*

